# Effects of Human Nucleolus Upon Guest Viral-Life, Focusing in COVID-19 Infection: A Mini- Review

**DOI:** 10.30699/IJP.2021.540305.2744

**Published:** 2021-12-15

**Authors:** Moslem Bahadori, Mohammad Hossein Azizi, Shahriar Dabiri, Neda Bahadori

**Affiliations:** 1 *Department of Pathology, Sina Hospital, Tehran University of Medical Sciences, Tehran, Iran*; 2 *Academy of Medical Sciences of Iran, Tehran, Iran *; 3 *Department of Pathology, Pathology and Stem Cells Research Center, Afzalipour Medical School, Kerman University of Medical Sciences, Kerman, Iran*; 4 *Tehran University of Medical Sciences, Tehran, Iran*

**Keywords:** Endotheliopathy, Nucleolus, Nucleolopathy, Viral infection

## Abstract

The nucleolus is a subcellular membrane-less structure of eukaryotic cells. In 1965, in a world’s southern summer summit in Uruguay, the role of the nucleolus as the site of ribosome synthesis, biogenesis, and processing of tRNA was conclusively established. Today, accumulating evidence confirm the multiple functions of the nucleolus, including tRNA precursor processing, cell stress sensing, as well as being influential in gene silencing, senescence, lifespan, DNA damage response (DDR), and cell cycle regulation. Therefore, nucleolopathy is observed in various human diseases. Modern advances have provided fundamental insights concerning how and why the nucleolus is targeted by different pathogenic organisms. Viruses are major organisms that disrupt the normal function of the nucleus and produce nucleoli proteins for facilitating the replication of viruses causing viral infections. In this review, we focus on the possible role of nucleoli upon coronavirus infections, particularly in coronavirus disease 2019.

## Introduction

During the 1830s, the nucleolus was first described in eukaryotic cells (1-3). Later, an Italian pathologist, Giuseppe Pianese, noticed its importance as showing excessive volume in malignant cells (4). However, the nucleolus function remained unknown until the 1960s, when it was recognized as the site of ribosome biogenesis and the center for protein homeostasis (5-10). Homeostasis of proteins (proteostasis) is composed of a group of coordinated cellular functions which ensure protein synthesis, folding, and degradation. These mechanisms are needed throughout the lifespan of an organism to maintain a functional proteome. The nucleolus is a multifunctional organelle and the prominent intranuclear part of eukaryotic cells (11-14). 

Apart from conventional nucleolar function, the nucleolus is involved in three essential fields, including viral replication, stem cell biology, and cellular senescence. Knowing distinct functions of the nucleolus, it is understandable that many replicating viruses in the nucleus interact with readily accessible nucleolar materials. As small obligatory parasites, viruses use the host materials to replicate and divert some of the cellular mechanisms for their own life. They alter the function of host cells to create a situation that favors their replication and functional activities (15-18). Nucleoli go through important morphological and behavioral modifications due to various viral infections. When viruses enter cells, they replicate using nucleolar ribosomal activity and nucleolar DNA, leading to cellular damage. We review the current research providing new information on nucleolar function in viral infections, especially coronavirus disease 2019 (COVID-19). 

Human Nucleolus and Viral Infections 

Nucleolar components and functions in health and disease have been the subject of research and discussion since the beginning of the 21^st^ century. The nucleolus is the hub center of ribosome biogenesis and a hotspot for polymerase-mediated transcription of RNA. Therefore, nucleoli are critical for protein synthesis, where rRNA is processed and transcribed, making a complex with the ribosomal subunits of the nucleolus. The nucleolus, as the regulator of cell cycling, cell differentiation, and cell stress response, has a great role in intracellular signaling. Nucleolar insufficiency may play a role in the nervous system degenerative pathologies, such as Alzheimer’s disease, cardiovascular dysfunction, or the malfunctions of other organs. The nucleoli have an important role in the regulation of autophagy and apoptosis. The remar-kable point is that viruses often hijack the nucleolus to support the growth of the transformed cell or enhance viral infection. This phenomenon of viral infection highlights recent advancements in the mechanistic un-derstanding of the interference of coronaviruses nucleo-protein with nucleolar antigen and host cells (17, 18). 

Viruses, as obligatory intracellular parasites, show complicated strategies for changing host cell function and creating a new environment for themselves. Generally, the viruses associated with specific diseases affect several human organs or tissues, some of which may be lethal (18). A common characteristic of viral nucleoproteins is localization at the nucleolus and interaction with nucleolus proteins. Viruses may enter the body in many ways depending on the type of viruses and vectors. Usually, the routes of entry in humans are surface epithelium, respiratory tract, alimentary tract, skin, eye, and genitourinary tract.

A typical infective cycle in the cell lysis process includes virus attachment to the cell surface using specific membrane receptors, traveling to cytoplasm across the plasma membrane, spreading into the cytoplasmic environment, producing viral RNAs, and proteins by genome replication. At the end of the cycle, the newly-formed viral components are assembled into virus particles. They are released from the infected cells and spread into new host cells. Nucleoli undergo important morphological modifications during cell infection. When the viruses interact with different cells or viral factors, many viral components traffic to and from the nucleolus. Numerous host nucleolar proteins are distributed in other components of cells or become modified, and some cellular proteins are relocated in the nucleolus of infected cells (2, 19-22). The interaction of the virus with the nucleolus is a pan-virus phenomenon during viral infection. The function outcome of host cell function after viral infection is variable. However, despite the variability in mechanisms, the common feature is the changing of nucleolar functions. Resear-chers observed that the majority of virus interactions with the nucleolus concern capsid structural proteins, which have some shared properties among all viral families. 

The first coronavirus outbreak as a severe acute respiratory syndrome (SARS) occurred in Guangdong province, China, in 2002 and 2003, with a total of 8,098 cases and 774 deaths worldwide. Another outbreak started as the Middle East respiratory syndrome (MERS) in 2012, affecting a total of 855 individuals and causing 333 deaths in 2014 with a 40% mortality rate (23). Later, in December 2019, there was a report from the city of Wuhan, China, of several patients initially diagnosed with pneumonia of unknown etiology. Epidemio-logically, the cases were linked to seafood (23). When the disease was proven to be caused by a coronavirus, it was named by the World Health Organization (WHO) in February 2020 as COVID-19 and the responsible pathogen has been identified as a new coronavirus (19). It was also cautiously named the 2019 novel coronavirus (2019-nCOv). This pandemic now has been re-named as SARS-CoV-2 by the International Committee of Taxonomy of Viruses (20). Entering the host cell, the main target of invader viruses is to disorganize protein synthesis, including altering nucleolar proteome machinery for their replication (22-26).

In eukaryotic cells, the nucleolus consists of over 700 proteins, which depending on their role, are grouped into separate classes (18). Fibrillarin and nucleolin are two major proteins of the nucleoli and are in charge of nucleolar assembly and the biogenesis of ribosomes acting as a chaperon for the intake of proteins into the nucleolus. The interaction of viral N-protein with nucleolin is a possible description of how the localiza-tion of coronavirus N-protein takes place in the nucleolus. This protein localizes to the nucleolus and is involved in the regulation of cell growth and cell cycle (27, 28). Accordingly, this finding provides fundamental insights into how and why the nucleolus is targeted by coronaviruses resulting in disrupted normal action and the production of nucleolar proteins to facilitate virus replication. Approximately 232 high-confidence protein interactions have been identified between SARS-CoV-2 protein and human proteins by researchers (29). They showed correlations between the replicate incidents of viral proteins. During SARS-CoV-2 infection, they observed changes in the expression of human proteins interaction in regards to their cell biology and anato-mical expression pattern (30). A group of researchers revealed that COVID-19 also expresses rogue antibody, autoantibody that attacks and blocks type 1 interferon, and protein molecules in the blood that have a critical role in fighting off viral infections (31). In addition, this protein is found to act as an immune defendant against coronavirus in the nucleolar proteome. 

COVID-19 Infection 

Coronavirus from the order of Nidovirales and the family of coronaviruses (COVs) belongs to the subfamily coronaviruses. It is composed of several generations, namely alpha, beta, gamma, delta, and lambda. This group of Nidovirales order is enveloped, non-segmented with the COV virions being spherical with a diameter of approximately 125 nm. The most common feature is the club-shaped spike projection emanating from the virion surface. Four major structural proteins, including Spike(S), Membrane (M), Envelop (E), and Nucleocapsid (N) proteins comprise the COV particle and all of them are encoded with the 3’ end of the viral genome (28). 

The genome of COV is a single-stranded positive-sense RNA and the genomes share a significant number of common features and functions, such as being highly conserved, expressing many nonstructural proteins, several unusual activities, and expressing downstream genes by the synthesis of 3’ nested sub-genome mRNA (29). The RNA genome is used as a template for the direct translation of the polyprotein. It encodes nonstructural proteins that form a double-membrane vesicle for the replication-transcription complex (30). The structural and accessory proteins are completely translated from the sgRNAs of COVs. Within the four Nidovirus families, the major differences are the number, type, and size of structural proteins with a significant alteration in the structure and morphology of their virions and nucleocapsid (29). Their role in the replication of COVs of many of the nonstructural (NSPs) and structural proteins has been reported. Although the functions of some of these NSPs are unknown, many of them have shown their definite roles. Four structural proteins are the key particles needed for virion assembly and the infection of COVs (30, 32, 33). 

Gordon* et al.* reported interactions between the proteins of SARS-CoV-2 and human host cell proteins, which are involved in several actual complexes and biological processes (34). Examples of these NSPs include DNA regulators (NSP1), epigenetic and genes expression regulators (NSP5, NSP8, and NSP13), and vesicle trafficking (NSP2, NSP6) proteins (2, 26). In different host cells and tissues, COVs display a wide range of tropism with alpha coronavirus and beta coronavirus usually infecting mammals (23, 24). A list of major pathogenic proteins of COVs has been reported by Cui* et al.*, which is valuable for a better understanding of the pathogenesis of COVs (35). The N-protein of COVs in the virus-infected cells can localize either the cytoplasm alone or the cytoplasm and nucleolus. Those N-proteins able to localize multiple signals require to determine their subcellular localization and then become functional. However, the N-protein of COVs commonly localizes the nucleolus, but nucleolar localization-/retention signals (NORSs) and pathways are not well understood. Localization usually requires a region of nucleoli with proteins rich in arginine residues and is likely cell cycle-dependent (36-38). 

COVID-19 and Endothelial Nucleolar Stress

By altering the integrity of vessel barriers, accumulating evidence suggests that the endothelial cell and its nucleolus activation and dysfunction participate in SARS-CoV-2 pathogenesis (26, 28-30, 32, 33). Endothelial cells of the lung parenchyma account for one-third of the cells in the lungs and act as the basic barrier between blood and lung interstitial spaces. They play a role in acute respiratory distress syndrome and other pulmonary disorders (33, 39, 40). The endothelium of vessels is a crucial interface between the blood flow and tissues and plays an important role with a series of notable properties that generally preserve homeostasis. The endothelial functions entail the control of blood flow, fibrinolysis, vasomotion, inflammation, and oxidative stress. Although they participate in regulating circulation and coordinating host defense mechanisms, they can contribute to disease when mal-functioning. 

**Fig. 1:**
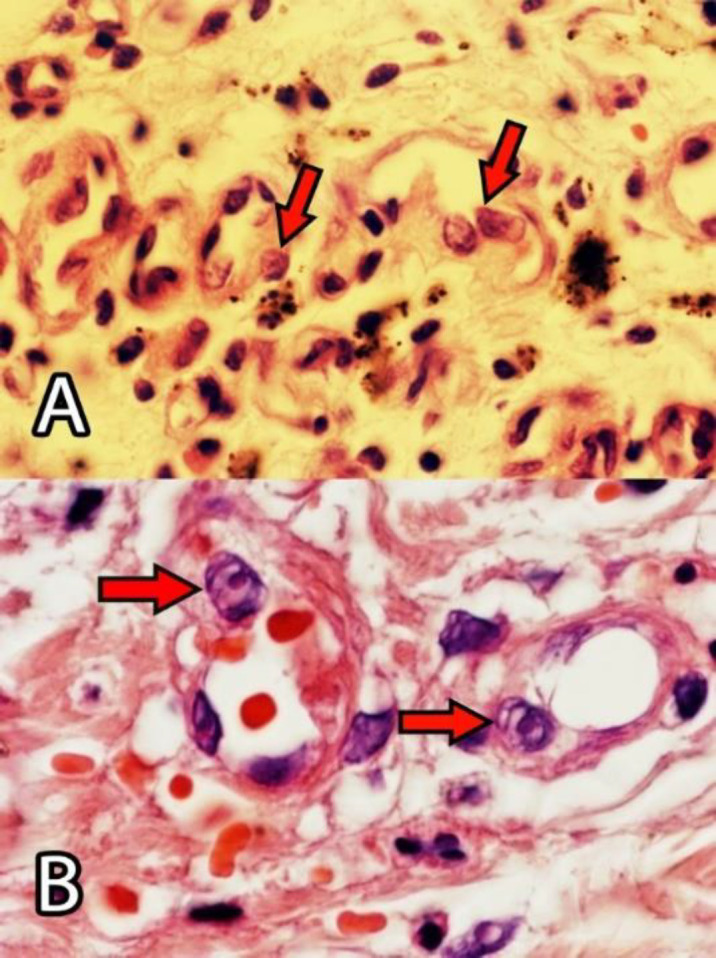
Swollen endothelial cells with prominent nucleoli of vessels in the alveolar interstitial space (A) and skin (B); (By Shahriar Dabiri MD)

**Fig. 2:**
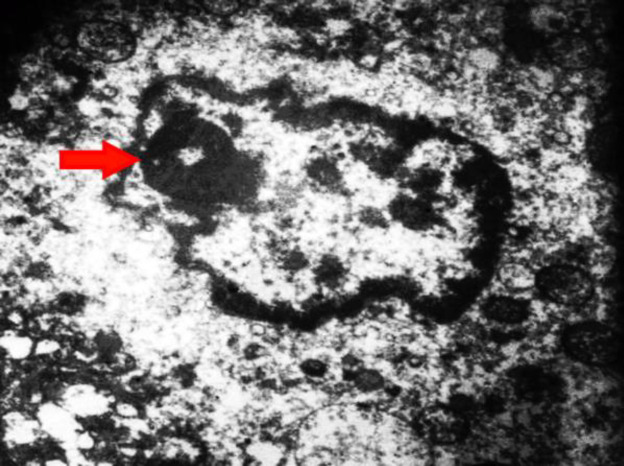
Electron microscopy showed the presence of nucleolus in the nucleus of diseased hepatocyte and swollen mitochondria; (Original magnification ×5,000) (Courtesy of Dr. Mitra Rezai, Shahid Beheshti University of Medical Sciences)

**Fig. 3:**
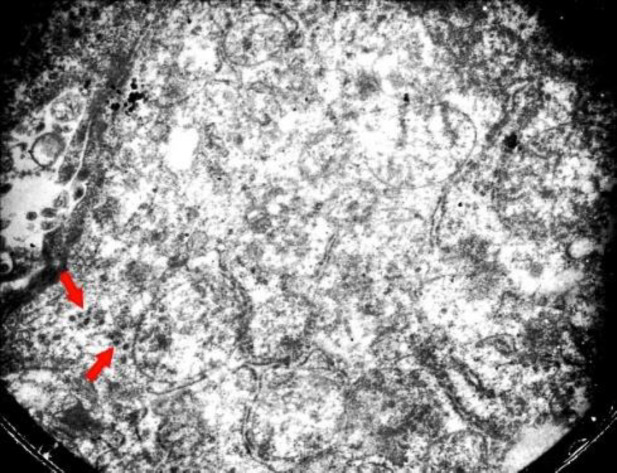
Electron microscopy showed the presence of virus particles in the cytoplasm and swollen mitochondria about 60 to 150 nm; (Original magnification ×15,000) (Courtesy of Dr. Mitra Rezai, Shahid Beheshti University of Medical Sciences)

**Fig. 4:**
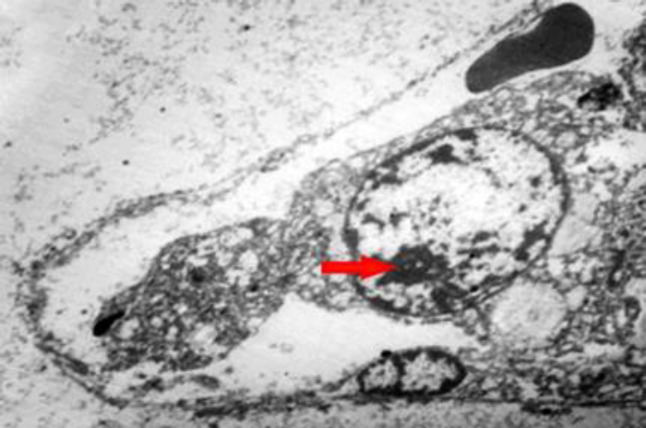
Electron microscopy showed the presence of nucleolus in the nucleus of swollen degenerated endothelial cells; (Original magnification ×1600). (Courtesy of Dr. Mitra Rezai, Shahid Beheshti University of Medical Sciences)

**Fig. 5:**
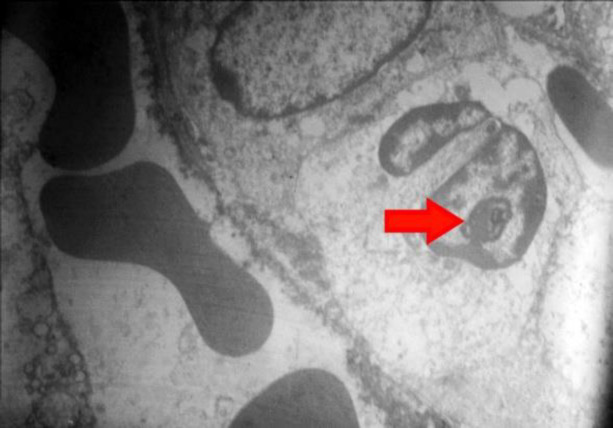
Electron microscopy showed the presence of nucleolus in the nucleus of neutrophil; (Original magnification ×5,000). (Courtesy of Mr. Majid Asadi PhD, Kerman University)

Concerning endothelial cells, COVID-19 infection causes a protean body of manifestations throughout the body ranging from head to toe, seemingly subverting indiscriminate havoc on multiple body organs, including lung, liver, kidney, heart, and GI tract. As a result, endothelial damage, particularly in the later complicated stages of COVID-19, represents an endothelial disease (31, 35, 39, 40). Apoptosis mediated by a virus may promote the disruption of endothelial cells and vascular barrier with interstitial edema and increase the activation of immune cells leading to widespread endotheliitis, the activation of platelets. Moreover, the coagulation cascade causes venous and arterial thrombosis (37). Endothelial cells under the stress of coronaviruses are a preferential target of COVID-19, resulting in wide-spread endotheliitis (33, 34, 38, 41-43). Postmortem examinations showed that the main focus of viral damages is the endothelial cells (44-46). We have observed endothelial damages in our autopsy cases in the endothelial cells (endotheliopathica), hepatocytes, neutrophils, and mitochondria (Figures 1-5) of involved organs (27). Therapies preventing vascular damages and medications that improve endothelial dysfunction, such as the inhibitors of angiotensin-converting enzyme, angiotensin receptor blockers, and statins may improve the outcome in COVID-19 (42). 

## Conclusion

Recently, researches demonstrated the critical role of nucleolus in viral infections, including coronavirus infections. The recent pandemic of COVID-19 provided much worldwide research on this subject, particularly the application of quantitative and analytical proteomics, as well as MCI (33, 41) that highlighted the value of systems biology approaches. These procedures elucidate the interactive biology and pathology of the nucleolus interfacing virus. Therefore, a better understanding of high mutual influence among nucleolar proteome and viral proteins is provided. The functional importance of the interface of these two components has been clarified. Recognition of viral mutations has particularly enabled us to ascertain the modification of viral-protein-nucleolar interactions, which is of great importance in evaluating these changes. In a recent publication, Rendeiro* et al.* searched for particular proteins in a multicenter study on the pathology of the lung affected by COVID-19 (31). They used multipurpose high-performance technologies, including MCI, to investigate the cellular composition and spatial architecture at the single-cell resolution of these human acute lung injuries. The authors focused on the expression of 36 proteins and found various nucleolar protein interactions with injured cells in many images. Accordingly, studies for evaluating mutational performances and validating the critical importance of nucleolar-viral interaction in these new cases, as well as demonstrating new approaches for either prevention or therapy should be the future target of an investigation. The emergence of sporadic cases and epidemics of new types of COVs are a severe global health threat. Changes in climate and ecology, as well as the increased interaction of humans with animals, may cause the outbreaks of new COV to be highly possible and unavoidable in the future. Consequently, there is an urgent need for sufficient and suitable health care accommodations. It is highly necessary to produce effective therapeutic agents and develop vaccines against COVs (30, 35). The final word is what Hiscox in the early 21^st^ century said that the nucleolar function is an important target for viral diseases (47).

## Authors’ Contribution

MB: Introduced the idea, collected data, preparing a draft of the manuscript.

MHA: Finalized the draft, data, and references.

Sh. D: Critically read the manuscript, prepare photographs, and add more data.

NB: Collecting data, editing and typing.

## Ethical Statement

 Not applicable.

## Conflict of Interest

The authors declared no conflict of interest.
